# Gastric Ulcers in Middle-Aged Rats: The Healing Effect of Essential Oil from *Citrus aurantium* L. (Rutaceae)

**DOI:** 10.1155/2012/509451

**Published:** 2012-11-01

**Authors:** C. M. Polo, T. M. Moraes, C. H. Pellizzon, M. O. Marques, L. R. M. Rocha, C. A. Hiruma-Lima

**Affiliations:** ^1^Department of Physiology, Institute of Biosciences, Botucatu, 18618-970, SP, Brazil; ^2^Department of Morphology, Institute of Biosciences, São Paulo State University (UNESP), Botucatu, 18618-970, SP, Brazil; ^3^Agronomic Institute of Campinas (IAC), Campinas, P.O. Box 28, 13012-970, SP, Brazil

## Abstract

The elderly population has experienced increased life expectancy as well as the increased incidence of gastric ulcers. The peels of fruits from *Citrus aurantium* L., popularly known in Brazil as orange bitter, are commonly used asatea form for the treatment of gastrointestinal tract disorders, such as ulcer and gastritis. We evaluated the healing effects of essential oil from the peels of *Citrus aurantium* fruits (OEC) on gastric ulcers in middle-aged rats. We examined the effects of a 14-day chronic OEC treatment on gastric mucosa in middle-aged male Wistar rats that were given acetic-acid-induced gastric lesions by morphometric and immunohistological analyses. Oral OEC treatment significantly reduced the lesion area (76%) within the gastric mucosa and significantly increased (*P* < .05) the height of regenerated mucosa (59%) when compared to the negative control group. Immunohistochemical analysis of the molecular markers such as COX-2, HSP-70, VEGF, and PCNA in the gastric mucosa confirmed that OEC treatment induced healing effects by increasing the number of new blood vessels and by augmenting gastric mucus in the mucosa glands. These results suggest that the oil from *Citrus aurantium* effectively heals gastric ulcers in middle-aged animals; however, safe use of OEC demands special care and precautions.

## 1. Introduction

An increase in life expectancy combined with a steady decline of the birth rate in developed countries has led to an unprecedented demographic revolution, characterized by an explosive growth in the number of elderly people [1]. Consequently, concerns about the health of the elderly has become more relevant; an increased life expectancy has paralleled an increase in the incidence of gastric and duodenal ulcers [2, 3]. Older individuals also tend to have a higher prevalence of comorbid factors, including *Helicobacter pylori* infection, smoking, presence of other diseases, or use of medications that increase their risk for acid-related disorders [4].

Previous studies have found a reduction of protective physiological factors in gastric mucosa, such as prostaglandins, mucus, and hormones, including serum gastrin, in the elderly population [5, 6]. Older individuals often have reduced acid secretion [7, 8], blood flow, and prostaglandin levels in the gastric and duodenal mucosa [9–12] as well as reductions in bicarbonate secretion [13] and mucosal cell proliferation [11, 14], among other conditions. According to Gabriel et al. [15], an individual over 60 years of age has a 3-fold increased risk of developing gastrointestinal complications after the use of nonsteroidal anti-inflammatory drugs (NSAIDs) compared to younger persons. Other drugs that are commonly used by the elderly, including tranquilizers, psychotropic drugs, diuretics, laxatives, antibiotics, and glucocorticoids, have harmful effects on the gastrointestinal tract [16]. Even some commonly antiulcerogenic drugs, such as proton pump inhibitors (lansoprazole and omeprazole), can cause acute nephritis [17] and hepatitis [18]. Other common adverse effects of proton pump inhibitors are nausea, diarrhea, constipation, and endocrine abnormalities, namely, gynecomastia [19]. Therefore, efficacious antiulcer drugs that do not present side effects are needed as well as therapies for the relief and healing of erosive lesions and the prevention of disease recurrence in younger and older populations.

*Citrus aurantium* L. (Rutaceae) is used worldwide to treat gastritis and other gastric disorders; its essential oil is commonly used as a flavoring agent. In Brazil, peels dried from *C. aurantium* named as “laranja-da-terra,” were commonly used asatea form to treat ulcers, gastritis, and stomachache and studies from Moraes et al. [20] already described the gastroprotective action of this essential oil to increase gastric mucus production in young rats and the dose-response curve as this essential oil characterized the dose of 250 mg/kg (p.o.) as the most effective. Therefore, we evaluated by morphometric and immunohistological analysis the ulcer healing effects of the essential oil from *Citrus aurantium* in middle-aged rats. 

## 2. Materials and Methods

### 2.1. Animals

Male Wistar rats aged 48 weeks and weighing between 485 and 730 g were randomly separated into three groups. Animals were obtained from the UNESP Central Animal House, Botucatu, and were fed a diet of certified Nuvilab (Nuvital, Brazil) with free access to tap water under standard temperature (22 ± 1°C) and 12 h dark-12 h light conditions. All experiments were performed in the morning and followed the recommendations of the Canadian Council on Animal Care [21]. The protocol was approved (July 2010) by the UNESP Institutional Animal Care and Use Committee (no. 231/CEEA).

### 2.2. Essential Oil

A sample of *Citrus aurantium* was collected (May 2008) in Botucatu, São Paulo, Brazil by Moraes and its exsiccates were deposited in the Herbarium “Irina D. Gemtchujnicov-” BOTU, Department of Botany at UNESP, under no. BOTU 23123. Essential oil was extracted from the fruit peels of *Citrus aurantium* L. by water vapor with the aid of a Clevenger-type device (Marconi, Brazil). Peels were mixed inside a glass balloon (5 L) with distilled water and put on a heated pad. The essential oil (OEC) obtained was stored in an amber bottle at 5°C until pharmacological experimentation and phytochemical analyses. 

### 2.3. Identification of Substances

The OEC samples were the same from obtained by Moraes et al. [20]. The sample was analyzed in a gas chromatographer coupled to a mass spectrometer (GC-MS, Shimadzu, QP-5000) operated by an electronic component (70 eV) equipped with a capillary column of fused silica (OV-5, 30 m × 0.25 mm × 0.25 *μ*m). Helium was used as the carrier gas (1.0 mL/min, White Martins, 99.9%), and the injector and detector were operated 24°C and 230°C, respectively, in *split* injection mode. Mass spectrums were acquired in the mass range of 40 to 500 m/z. The OEC (1 *μ*L) was diluted in ethyl acetate (1 mL, chromatographic grade) before injection of 1 *μ*L of sample at a 1 : 15 split ratio. The column temperature was heated to 60°C and programmed at 3°C/min to 240°C. Chemical identification was performed by comparison of mass spectra with the GC-MS system database (NIST 62 lib.), the literature, and with the Kovats retention indices [22]. The exact chemical composition of essential oil from fruit peels of *Citrus aurantium* was measured and already described by Moraes et al. [20] that indicated the presence of limonene (79.83%), myrcene (1.43%), and octanal (0.45%). 

### 2.4. Effects on Acetic-Acid-Induced Gastric Lesion

The experiment was performed according to the method described by Takagi et al. [23] with some modifications. Three groups of middle-aged Wistar rats (48 weeks) were starved for 12 h (*n* = 14 to 15). Under anesthesia, a laparotomy was performed through a midline epigastric incision. After exposing the stomach, 0.05 mL (v/v) of a 30% acetic acid solution was injected into the subserosal layer in the glandular portion of the anterior wall. The stomach was bathed with saline (20°C) to avoid adherence to the external surface of the ulcerated region. The abdomen was then closed, and all of the animals were fed normally. We selected the lowest effective dose of OEC (250 mg/kg, body wt) to evaluate the healing effect of *C. aurantium* based on data from Moraes et al. [20] that already realized dose-response curve from this essential oil. A separate group was treated with 100 mg/kg of cimetidine (19.1% pure, Sigma Co., U.S.A.) or vehicle (10 mL/kg at Tween 80; 0.8%). All treatments were administered orally once daily for 14 consecutive days beginning one day after surgery. One day after the final drug administration, the rats were sacrificed, and their stomachs were gently removed. Body weight was recorded daily throughout the experiment, macroscopic analysis was performed, and the weights of some organs such as liver, kidneys, heart, spleen and lung were determined. The all relative organ weights (organ weights relative to body weights) were transformed in arcsine before statistical analysis. Gastric lesions were evaluated by examination of the inner gastric surface with a dissecting magnifying glass. The macroscopic ulcer area (mm^2^) was determined as described by Takagi et al. [23]. *Biochemical analysis: *Blood samples were collected immediately after the sacrifice and submitted to centrifugation (3000xG for 10 min). After the centrifugation, the resulting serum was frozen at −20°C until biochemical analysis was performed. Biochemical markers, including glucose, urea, creatinine, aspartate aminotransferase (AST), and alanine aminotransferase (ALT), were measured in the serum by an automated biochemical analyzer (SBA-200, CELM, Brazil).* Histological methods: *Stomach lesions were located, sectioned, and fixed in ALFAC solution (85% alcohol, 5% acetic acid, and 10% formaldehyde) for 24 h at 4°C. Samples were then embedded in Paraplast and cut into 7 *μ*m thick sections that were stained with periodic acid-Schiff (PAS) [24] and hematoxylin and eosin (H&E) [25]. Paraffin slides were processed for H&E staining and immunohistochemical reaction in blood vessels. We used a minimum of 6 fields for analysis, and the results were statistically analyzed. *Morphometric analysis*: A section of stomach was examined using a Leica microscope coupled with Leica Qwin Software (Leica-England). The height of regenerated mucosa was measured (*μ*m) by employing a variation of the method used by Ishihara and Ito [26]. *Immunohistochemical analysis:* Representative sections were deparaffinized, rehydrated, and immunostained using the ABC method. Nonspecific staining was blocked with H_2_O_2_ and goat serum prior to incubation with the antiserum. After a wash in phosphate buffered saline (PBS 0.01 mol/L, pH 7.4), the sections were incubated in secondary antiserum (ABC kit) and again washed with PBS. Finally, the ABC complex was prepared, and the reaction was performed using DAB solution (3,3′-diaminobenzidine-tetrahydrochloride) containing 0.01% H_2_O_2_ in PBS. After immunostaining, the sections were lightly counterstained with Mayer's hematoxylin, and the immunoreactive cells were observed under a Leica microscope coupled with Leica Qwin Software (Leica-England). For the control reactions, a group of slides were processed without the primary antibody, and the primary and secondary antibodies were omitted from a second group of slides. Staining was performed with antibodies against PCNA (Novo Castra), HSP-70 (Santa Cruz SC 1060), COX-2 (Cayman Chemical), and VEGF (Santa Cruz SC 7269). 

### 2.5. Statistical Analysis

Results are expressed as the mean ± SEM. Statistical significance was determined by ANOVA one-way analysis of variance followed by a Dunnett's post hoc test with the significance level set to *P* < 0.05. 

## 3. Results and Discussion

 It is well known that gastric ulcers occur more frequently among persons more than 60 years of age ([27], and it estimated that by 2020, more than 16% of the United States population will be older than 65 years of age [4]. According to Del Vecchio Blanco et al. [27], the size of gastric ulcers in older patients is appreciably larger than in younger individuals due to deficiencies in the healing process. 

 In the present study, we used an acetic-acid-induced gastric ulcer model because it accurately reflects human peptic ulcer disease [11]. Application of acetic acid to the gastric mucosa in middle-aged rats quickly results in ulcerated areas. After 14 consecutive days of oral treatment with OEC, healing of the gastric mucosa was markedly accelerated (Table 1). 

Oral OEC treatment (250 mg/kg/day) significantly reduced the lesion area by 76% compared to the control group, and cimetidine treatment (100 mg/kg/day) promoted only a 23% cure rate compared to the control group (*P* > 0.05). Our preliminary data show that young rats (8-week old) treated with OEC (50, 100, and 250 mg/kg) provided significant gastric protection at doses of 250 mg/kg against different ulcerogenic agents and at the same dose, this OEC also has a healing action by reducing gastric lesions (44%) [28]. Tsukimi and Okabe [29] observed that the quality of gastric ulcer healing differs between young and middle-aged rats. Rats aged 48 weeks treated with two different anti-ulcer agents (cimetidine and omeprazole) presented a higher ulcer index than 8-week-old rats [26]. Therefore, our results show that 14-day OEC treatment exerts greater gastric ulcer healing in middle-aged rats than in young rats. 

**Figure 1:**
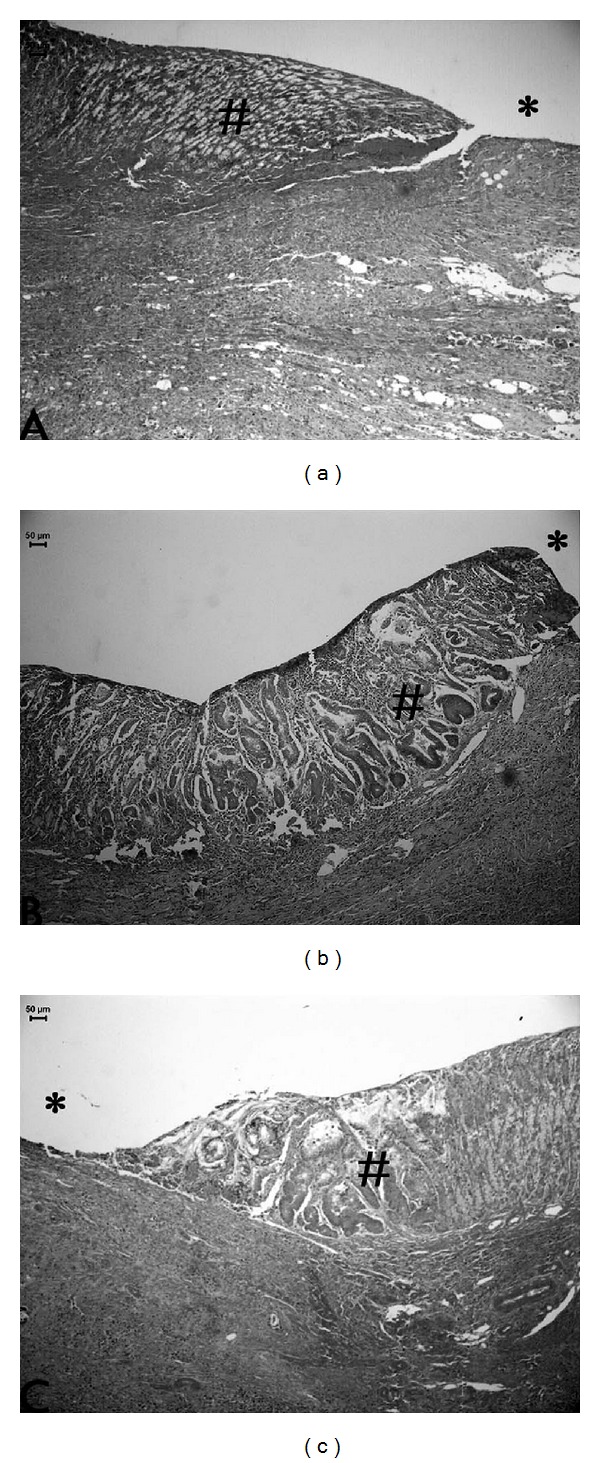
Photomicrography of histological sections of stomach lesions produced by acetic acid in middle-aged rats stained with H and E. Note the area of reorganization of board of lesion indicated by (#) and lesion area*. Vehicle (a), cimetidine (b), and *Citrus aurantium* (c). Asterisks indicate lumen of stomach in area with lesions induced by acetic acid in rats. Microscopy magnification 160x.

The healing effects of OEC and cimetidine were also observed by histological analyses (Figure 1, Table 1). Our results also proven by histological photomicrography the increase in height of reepithelization mucosa by middle-aged rats treated with OEC. The regenerative area increased 59% and 46% by treatment with cimetidine and OEC, respectively, when compared to the control group without treatment (*P* < 0.05). These results suggested that OEC stimulated some proliferative factors in gastric mucosa that were important factors to regeneration of injured gastric mucosa.

**Figure 2:**
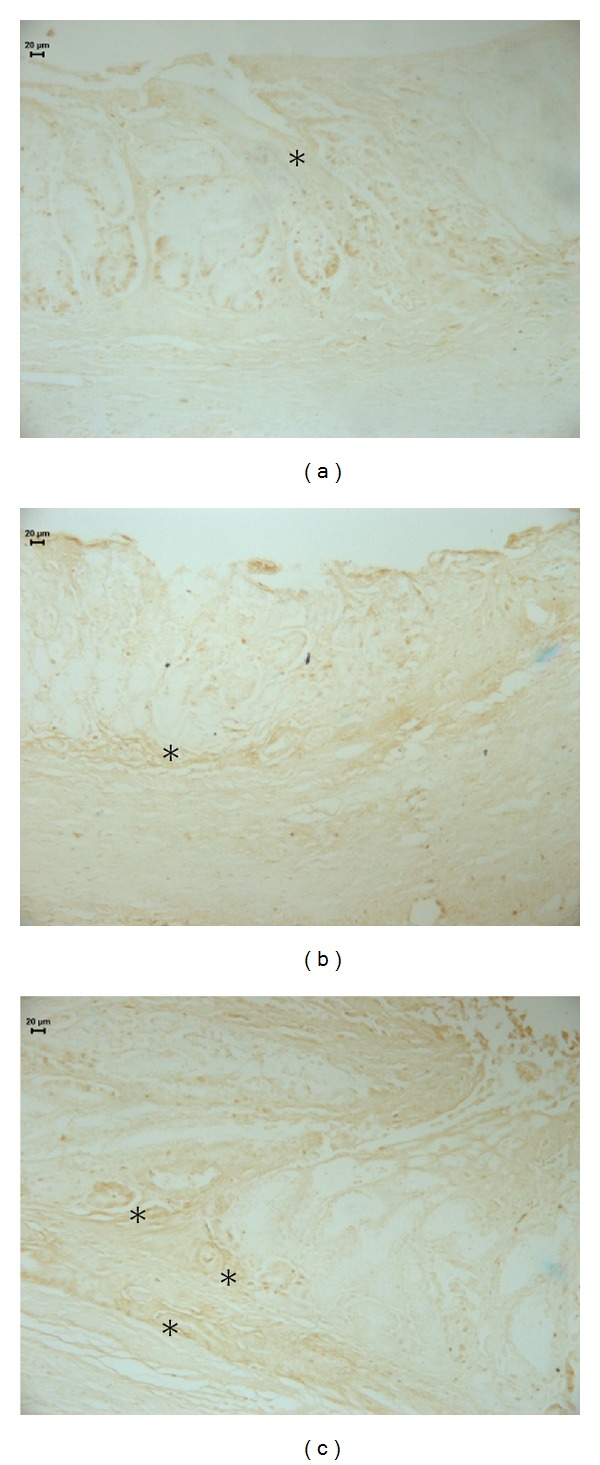
Photomicrography of histological sections of stomach lesions produced by acetic acid in middle aged rats using the immunolabeling by PCNA. Note the cells PCNA in (*). Vehicle (a), cimetidine (b), and *Citrus aurantium* (c). Microscopy magnification 640x.

According to Tarnawski et al. [30], the ulcer re-epithelialization is essential process for gastrointestinal ulcer healing and without restoration of a continuous epithelial barrier and the mucosa would be vulnerable to mechanical or chemical injury and infections thereby preventing ulcer healing. Cellular proliferation plays an essential role in maintaining the integrity of the gastric mucosa and an important proliferation marker is PCNA (proliferating cell nuclear antigen), a highly conserved 36 kDa nuclear peptide that is expressed during cell proliferation [31]. PCNA immunohistochemical analysis (Figure 2, Table 2) revealed that only the vehicle-treated group presented more proliferating cells in the stomachs (*P* < 0.05) than the cimetidine and OEC-treated groups.

 Some studies described that the reduction of neutrophils infiltration into ulcerated gastric tissues has been implicated in the promotion of healing of acetic-acid-induced chronic ulcer in rats [32]. Taken together, our results suggest that OEC treatment promoted accelerated healing of gastric mucosa in middle-aged rats when compared to the control group, in which cells proliferated during the later phase and lesion area was larger than OEC. Using PCNA immunostaining, Polo et al. [28] observed that OEC treatment in young (8-week old) rats during 14 days promoted intense cell proliferation in the lesion area of young animals, and the healing process was still ongoing, even at the end of the 14-day treatment. These findings indicate that OEC treatment promoted gastric ulcer healing in both middle-aged and young rats. However, OEC provided a faster healing rate and higher cure ratio in the middle-aged compared to the young rats based on rise regeneration area of OEC group. 

**Figure 3:**
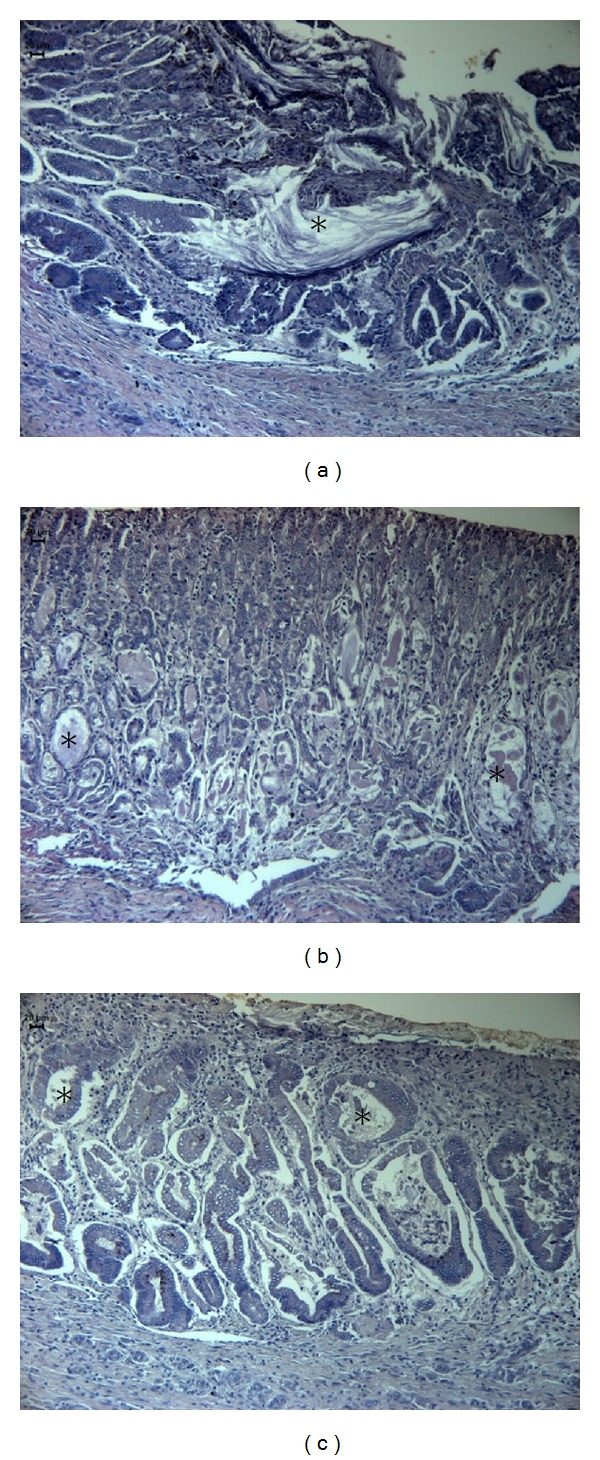
Photomicrography of histological sections of stomach lesions produced by acetic acid in middle-aged rats stained with PAS. Note the secretion inside of glands (*) and the increased of lumen diameter in special C group treated with *Citrus aurantium*. Vehicle (a), cimetidine (b), and *Citrus aurantium* (c). Microscopy magnification 640x.

Laine et al. [32] showed that mucus strengthens the mucosal barrier. For the healing process, it is desirable that mucus strength be augmented or at least maintained to protect the regenerating gastric epithelium. PAS staining (Figure 3(c)) showed increased mucus production after 14 days of OEC treatment compared to the saline group (Figure 3(a)). We measured the size of mucus glands within oxyntic glands and found that OEC treatment increased mucus production and swelling of the glands (Figure 3(c)), whereas middle-aged rats treated with vehicle showed glands that were less dilated and had little or no mucus (Figure 3(a)). Morphological analysis also revealed a difference in the organization of the mucosa tubular glands in OEC group, which was positively correlated with ulcer healing. This result was not surprising because the mucus layer protects newly formed cells against damage caused by acidic pH and proteolysis from gastric secretions [33, 34]. 

**Figure 4:**
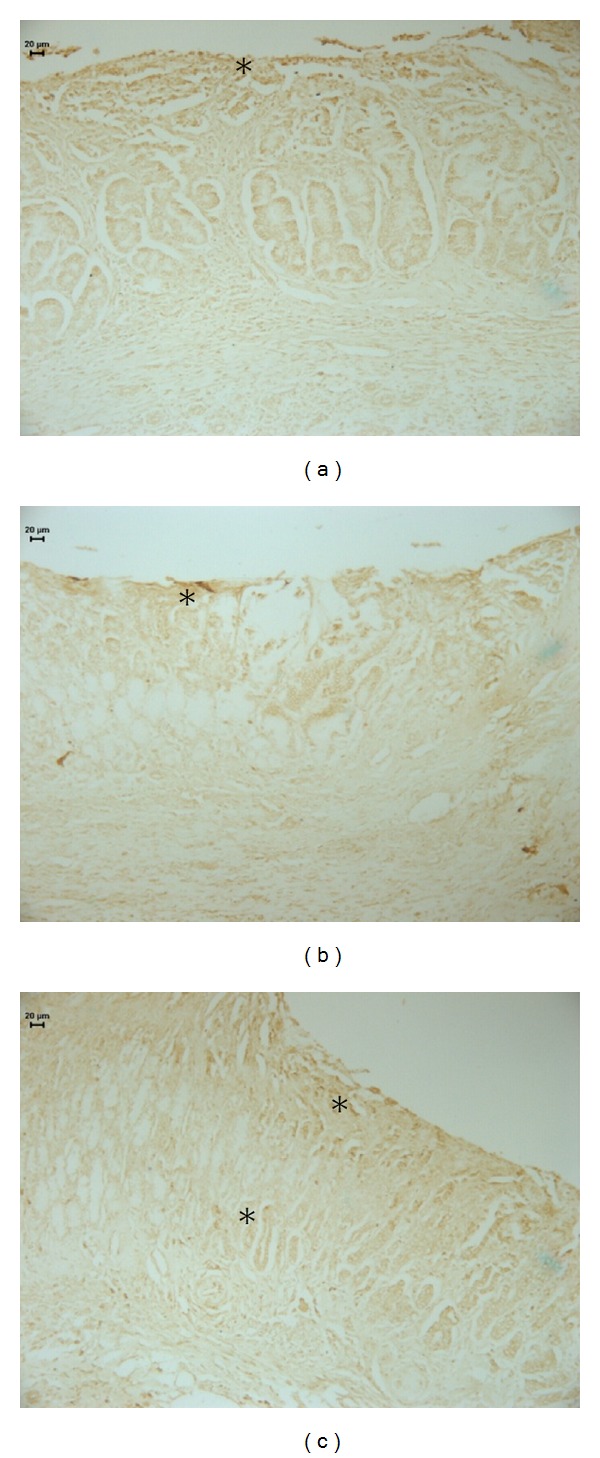
Photomicrography of histological sections of stomach lesions produced by acetic acid in middle-aged rats using the imunolabeling by HSP-70. Note the area HSP-70 positive in (*). Vehicle (a), cimetidine (b), and *Citrus aurantium* (c). Microscopy magnification 640x.

Heat shock proteins (HSPs) conduct the folding, assembly, and transport of proteins and protect cells from the cytotoxic effects of aggregated proteins produced during stress [35]. Our results demonstrate that middle-aged control-treated rats expressed a higher amount of HSP-70-positive cells than rats treated with cimetidine or OEC (Figure 4, Table 1). 

According to Tsukimi and Okabe [29], HSP-70 is expressed in proliferating cells during the re-epithelialization phase of the lesion healing process after, according to our findings, the proliferative phase in the OEC group. Therefore, during the proliferative phase, rats treated with cimetidine or OEC do not express HSP-70.

 Besides HSPs, COX-2 is another important factor for gastric mucosal defense. This enzyme rapidly responds to various proinflammatory stimuli, such as mitogens, hormones, and cytokines, and is thought to be responsible for PG production at the inflammatory site. COX-2 induces the production of several growth factors, including VEGF, and has an important role in tissue repair [36]. The expression of COX-2 is higher in the ulcerative margins and disappears at regions close to the cicatrisation [37].

**Figure 5:**
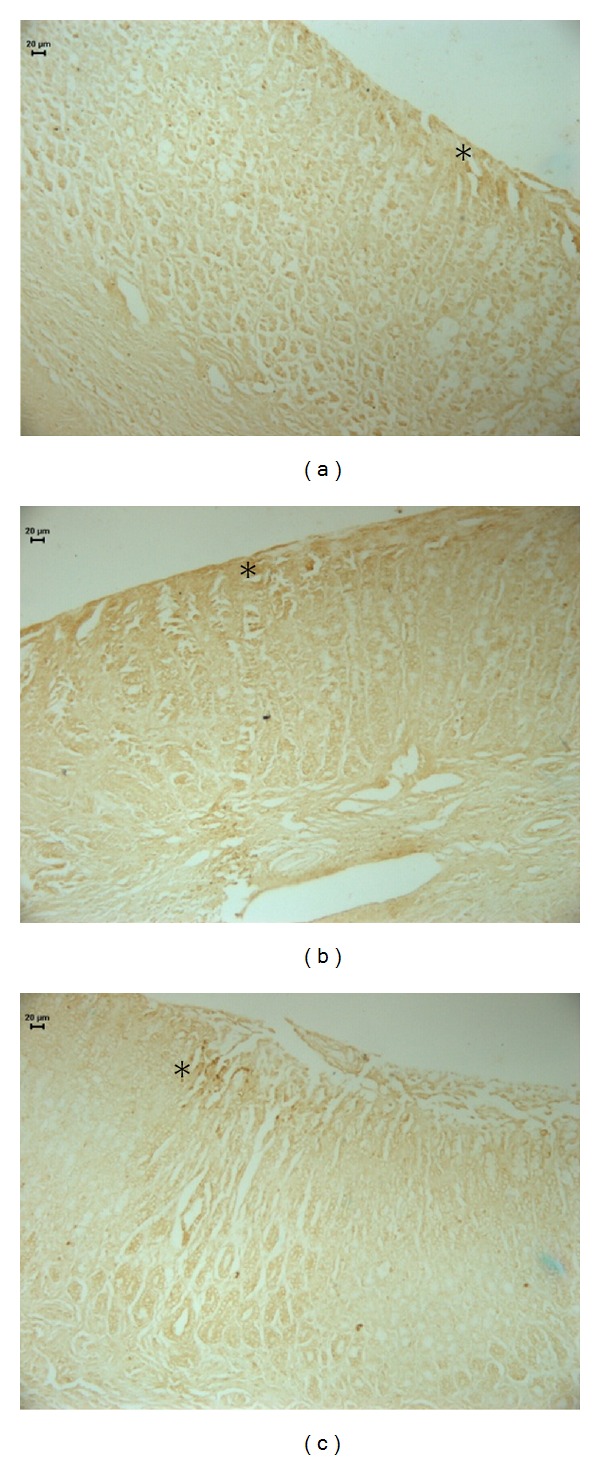
Photomicrography of histological sections of stomach lesions produced by acetic acid in middle-aged rats using the immunolabeling by COX-2. Note the area COX-2 positive in (*). Vehicle (a), cimetidine (b), an *Citrus aurantium* (c). Microscopy magnification 640x.

 Our data (Table 2, Figure 5) show no significant difference in the number of labeled cells expressing COX-2 in any of the treatments (*P* > 0.05). 

However, a previous study [34] in which young rats (8-week old) were treated with the same OEC found that COX-2 expression increased after 14 days. The time course of COX-2 expression is consistent with the plenary phase of cell proliferation (PCNA). This finding is in contrast to middle-aged rats, in which this stage had already passed after 14 days of OEC treatment, as indicated by a smaller gastric lesion area. These results corroborate previously described analyses of VEGF expression.

Wallace and Devchand [38] reported that reconstruction of the entire structural architecture of the damaged gastric mucosa may require several weeks and involves the formation of granulation tissue at the base of the ulcers, formation of new vessels (angiogenesis), and reestablishment of the entire glandular architecture. Angiogenesis is critical for the improvement of gastric mucosa and the prevention ulcer relapse [39]. One technique to measure the formation of new vessels is to determine VEGF (vascular endothelial growth factor) expression in endothelial cells and platelets [40]. Szabo et al. [41] described the role of VEGF to accelerate the healing of chronic gastric ulcers, chronic erosive gastritis, and ulcerative colitis but reported little or no acute gastric protection. 

**Figure 6:**
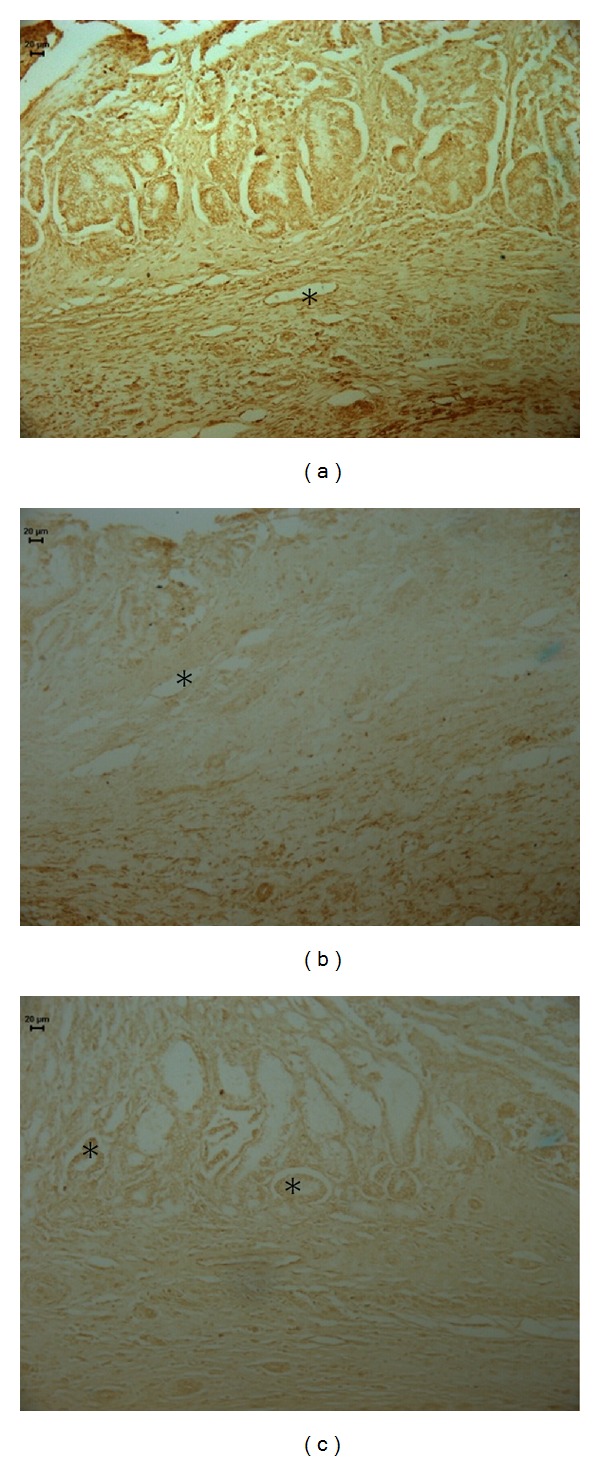
Photomicrography of histological sections of stomach lesions produced by acetic acid in middle-aged rats using the imunolabeling by VEGF. This imunolabeling evidences the vessel indicated in (*). Vehicle (a), cimetidine (b), and *Citrus aurantium* (c). Microscopy magnification 640x.

 Our results show that VEGF expression in the gastric mucosa of middle-aged rats treated with OEC was 3-fold higher than in the control group (Table 1 and Figure 6). Increases in of the number of new vessels in the OEC group (*P* < 0.001) contributed to increased thickness and the quality of healing. In a study of ulcer healing in young rats [20, 28], OEC treatment did not produce significant VEGF expression in the gastric mucosa. This result indicates a delay in the healing phase of young rats treated with OEC compared to senescent animals that received the same treatment. 

Over twenty years ago, Garner [42] predicted that the future of anti-ulcer drug research must address the multifactorial etiologies of gastric ulcer as well as attempt to cure the disease rather than simply induce antisecretory activity. However, new drugs must be efficacious and safe for human consumption. Our results show that in each treated group the body weight between the first and last day of treatment decreased; however, this decrease was not significant when compared to the result in the control group (*P* > 0.05). The safety of OEC was supported by the lack of mortality throughout the 14 consecutive days of treatment. The analysis of some organs (Table 3) revealed a significant increase in the weight of liver and kidney in the OEC-treated group compared to the control group (*P* < 0.01). This OEC-induced enlargement of organs was not confirmed by the biochemical marker (AST or ALT) analysis. Furthermore, the expression of other biochemical enzymes was not significantly altered. There was no significant difference in the biochemical analysis of serum between the treatment groups (Table 3). Because there was no indication of hepatic or renal toxicity, perhaps the metabolization of oil compounds overloaded the organs (liver and kidneys), but not enough to cause damage. It is important and necessary to use another marker of toxicity, such as sorbitol dehydrogenase (SDH), an enzyme marker of hepatotoxicity that is more selective for liver damage than AST or ALT because its age-related decline in activity is less than those of other marker enzymes [43]. 

Through the results of this study, the essential oil from *Citrus aurantium*, administered at the dose of 250 mg/kg for 14 consecutive days, significantly reduced the lesion area of gastric mucosa in middle-aged rats and significantly increased the height of regenerated gastric mucosa. Immunohistochemical analysis of gastric mucosa confirmed that OEC induced healing activity by increasing the formation of new blood vessels (VEGF) and by augmenting the gastric mucus present in the mucosa glands (PAS). These results suggest that the essential oil from *Citrus aurantium* is effective at healing gastric ulcers in middle-aged rats; however, its safety in humans must be thoroughly evaluated. 

## Figures and Tables

**Table 1:** Effect of 14-day treatment with essential oil (OEC) from *Citrus aurantium* (250 mg/kg) on healing the acetic acid-induced ulcer in the stomachs of middle-aged rats.

Treatment (p.o.)	Dose (mg/kg)	Macroscopic analyses		Histological analyses	
		Lesion area (mm^2^)	Curative ratio (%)	Regeneration area (*μ*m)	Curative ratio (%)
Negative control	—	1.67 ± 0.42	—	256.07 ± 20.67	—
Positive control	100	1.29 ± 0.50	22.75	375.00 ± 33.21*	46.44
OEC	250	0.40 ± 0.18*	76.05	407.87 ± 28.48**	59.28

(*n* = 14-15). Results are mean ± SEM. ANOVA followed by Dunnett's test. *P < 0.05; **P < 0.01.

**Table 2:** Counting of cells stained with specific antigen and neutrophils in the tissue regeneration of gastric ulcer induced by acetic acid in middle-aged rats (aged 12 months) after treatment with essential oil (OEC) of *Citrus aurantium*.

Treatment (p.o.)	Doses (mg/kg)	Neutrophils (cells/mm^2^)	Cells stained with specific antigen			
			PCNA	HSP-70	COX-2	VEGF
Negative control	—	1.82 ± 0.29	6.67 ± 0.32*	10.23 ± 1.19**	2.54 ± 0.49	2.87 ± 0.30
Positive control	100	1.57 ± 0.20	5.30 ± 0.40	4.86 ± 0.43	2.44 ± 0.29	3.08 ± 0.40***
OEC	250	1.38 ± 0.27	4.17± 0.30	7.40 ± 0.43*	3.15 ± 0.47	6.07 ± 0.33***

Results are mean ± SEM. ANOVA followed by Dunnett's test (*n* = 14-15). *P < 0.05, **P < 0.01, and ***P <0.001.

**Table 3:** Evaluation of toxicity in middle-aged rats of the essential oil (OEC) obtained from peels of *Citrus aurantium* after 14 consecutive days of treatment.

Treatment (p.o.)		Control (10 mL/kg)	Cimetidine (100 mg/kg)	OEC (250 mg/kg)
Weight (arcosine)	Kidney	0.52 ± 0.04	0.51 ± 0.15	0.57 ± 0.05*
	Lungs	0.27 ± 0.19	0.33 ± 0.14	0.30 ± 0.16
	Liver	2.03 ± 0.14	2.07 ± 0.61*	2.32 ± 0.20**
	Heart	0.27 ± 0.05	0.28 ± 0.03	0.28 ± 0.06
	Spleen	0.14 ± 0.03	0.16 ± 0.03	0.15 ± 0.03

Biochemistry	Urea (mg/dL)	30.57 ± 4.29	34.13 ± 6.44	28.47 ± 3.40
	Creatinine (mg/dL)	0.76 ± 0.13	0.71 ± 0.08	0.75 ± 0.12
	AST (UI/L)	165.46 ± 30.59	180.73 ± 18.10	173.36 ± 33.23
	ALT (UI/I)	38.42 ± 4.32	35.80 ± 6.35	34.13 ± 5.87
	Glucose (UI/L)	79.57 ± 9.74	77.80 ± 9.49	76.87 ± 9.69

Results are mean ± SEM. ANOVA followed by Dunnett's test. (*n* = 14-15), *P < 0.05, and**P < 0.01.

## Abbreviations

ALT: Alanine aminotransferase
AST: Aspartate aminotransferase
COX-2: Cyclooxygenase 2
H&E: Hematoxylin and eosin
HSP-70: Heat shock protein-70
NSAIDs: Nonsteroidal anti-inflammatory drugs
OEC: Essential oil from the peels of *Citrus aurantium* fruits
PAS: Periodic acid-Schiff
PCNA: Proliferating cell nuclear antigen
VEGF: Vascular endothelial growth factor.
